# The effect of the irrigant activation protocol on postoperative pain in maxillary incisors with asymptomatic apical periodontitis: A three‐arm randomized clinical trial

**DOI:** 10.1002/cre2.786

**Published:** 2023-10-03

**Authors:** Mohammad Tamer Abbara, Samar Akil, Omer Hamadah, Hassan Achour, Ghina Mahayni, Yasser Alsayed Tolibah

**Affiliations:** ^1^ Department of Endodontics, Faculty of Dentistry Damascus University Damascus Syria; ^2^ Department of Oral Medicine, Faculty of Dentistry Damascus University Damascus Syria; ^3^ Faculty of Dentistry Al‐Sham Private University Damascus Syria; ^4^ Department of Pediatric Dentistry, Faculty of Dentistry Damascus University Damascus Syria

**Keywords:** diode laser, irrigation activation systems, passive ultrasonic activation, XP‐Endo Finisher

## Abstract

**Objectives:**

This study aimed to compare the effects of three irrigation activation systems (IAS) on postoperative pain (PP) in activating three final irrigants: sodium hypochlorite 5.25%, ethylenediaminetetraacetic acid 17%, and chlorhexidine 2%.

**Materials and Methods:**

This parallel randomized clinical trial included referred patients with asymptomatic large‐sized apical lesion incisors. A standard method was followed in the canal cleaning and shaping for all included patients in the study. Then, the patients were randomly assigned (1:1 allocation) into three groups: G1 (*n* = 20) with passive ultrasonic irrigation activation; G2 (*n* = 20) with XP‐Endo Finisher file activation; and G3 (*n* = 20) with diode laser (810 nm) activation. PP was estimated in all groups using a visual analog scale after 1, 3, 7, and 14 days of treatment. Comparisons between the groups were made using the Kruskal−Wallis test, whereas the Mann−Whitney *U* test was used in the pairwise comparisons.

**Results:**

Sixty patients were followed‐up in this trial. There were significant differences between the groups in terms of PP After 1, 3, and 7 days of treatment (*p* = 0.002, *p* = 0.017, and *p* = 0.006, respectively). On the first day of treatment, G3 showed the lowest PP compared with G1 and G2 (*p* = 0.007 and *p* = 0.001, respectively). On the third day of treatment, G3 showed less PP compared with G2 (*p* = 0.005). On the seventh day of treatment, G2 showed the highest PP compared with G1 and G3 (*p* = 0.012 and *p* = 0.003, respectively).

**Conclusions:**

The XP‐Endo Finisher file caused the highest PP level especially in the next day and 3 days of the treatment, whereas the diode laser had the lowest PP level during the first week of treatment. It is noteworthy that PP disappeared completely after 2 weeks of treatment with all three IASs.

**Trial Registration:**

The trial was registered in the ISRCTN registry (Trial ID: SRCTN99457940).

## 1 INTRODUCTION

Root canal treatment commonly affects periodontal tissues either mechanically by files‐shaping procedures (Bürklein & Schäfer, 2012) or chemically by irrigation procedures (Almeida et al., 2012), and, thus, can cause postoperative pain (PP), with a prevalence between 3% and 58% (Sathorn et al., 2008). Chemical cleaning by irrigant solutions plays an important role in excluding necrotic pulps, canal debris, bacteria, and their remnants, especially in areas where mechanical instruments cannot reach within the root canal system (Versiani et al., 2016). Sodium hypochlorite (NaOCl), ethylenediaminetetraacetic acid (EDTA), and chlorhexidine (CHX) have been widely used as final irrigants during necrotic teeth treatment (Prada et al., 2019). It was also mentioned in the endodontic literature that the use of various irrigation activation systems (IAS) had improved the effectiveness of irrigation solutions and enhances their penetration to the entire length of the root canal (Gu et al., 2009). NaOCl is considered the most commonly used irrigant because of its antimicrobial and antibiofilm properties and organic tissue dissolution capacity (Haapasalo et al., 2014). Several studies showed that activating NaOCl by various IASs had improved its ability to cleaning of biofilm‐infected dentine, improve its access to the apical third of the canal and clean the lateral canals, reduce bacterial load, and thus shorten irrigation time (de Gregorio et al., 2010; Ordinola‐Zapata et al., 2014; Pasqualini et al., 2010).

EDTA 17% solution is commonly implemented in smear layer removal caused by mechanical cleaning (Violich & Chandler, 2010). This layer can reduce the effect of the intra‐canal medicament and affect the sealing of the root canal obturation (Violich & Chandler, 2010). It can also harbor bacteria that can multiply and penetrate the dentinal tubules (Haapasalo et al., 2014). Moreover, diffrent IASs had enhanced the ability of EDTA in smear layer removal, and increased the ability of NaOCl and EDTA combination in reduction of *Enterococcus faecalis* (Neelakantan et al., 2015; Rasheed & Jawad, 2021). CHX is a bactericidal agent that can kill microorganisms in less than 30 s as an aqueous solution (Vianna et al., 2004). Moreover, CHX has a prolonged antimicrobial efficiency due to its substantivity (Vianna & Gomes, 2009). In addition, the study of Salas et al. (2021) showd that the activate of CHX 2% using different IASs had increased its penetration into dentinal tubules of three‐thirds of the root canal.

The final irrigation of necrotic teeth could have a logical sequence (Peters et al., 2021), it is beginning with NaOCl, which works to organic tissues in addition to its disinfectant properties (Zehnder, 2006), then the irrigation with a chelating irrigant to remove the smear layer from the dentinal walls of canal and dissolve the inorganic tissues to increase the penetreation of the dentinal tubes (Violich & Chandler, 2010). Finally, CHX is irrigated to enter the dentinal tube due to its long‐term action against microorganisms (Prada et al., 2019), and it is worth mentioning that using CHX as the final irrigant in can also relieve the PP in the necrotic teeth (Bashetty & Hegde, 2010). Moreover, irrigant solution is separated from each other using saline to prevent the unfavorable interacting and the long‐term harmful effects of some irrigants (Prada et al., 2019).

Passive ultrasonic irrigation (PUI) has a series of advantages: it enhances removal of the pulp and dentin debris, has great efficiency in removing bacteria compared with manual agitation, and effectively cleans curved canals and isthmuses (Van der Sluis et al., 2007). PUI relies on sending acoustic energy through ultrasonic waves through a file or smooth oscillating wire to the irrigation solution to induce two actions (Van der Sluis et al., 2007): the first is acoustic streaming, which defines as generating a hydrodynamic force and dynamic pressure within the irrigant, and the second is cavitation, which is the formation of ultrasonically generated vapor‐containing bubbles within a fluid (Terauchi et al., 2022). Moreover, a combination of PUI with NaOCl, EDTA, and CHX irrigants has been considered the most effective irrigants for the elimination of *E. faecalis* (Prada et al., 2019). The XP‐Endo Finisher file is a non‐tapered nickel−titanium #25 file (XP‐Endo Finisher; FKG) made from an alloy that is affected by body temperature; the alloy is in the Martensite phase when it is cooled, and, as a result, the file is straight. When the file is exposed to body temperature, it changes to the Austenite phase and expands or folds for an equivalent size. This phase‐changing feature will help to eliminate the bacteria and debris located in the irregularities of the root canal system (Trope & Debelian, 2015). Moreover, XP‐Endo Finisher agitation has been widely used as an IAS to remove accumulated hard‐tissue debris, the smear layer, and microbes from the root canal system (Ballal et al., 2020). Laser technology has many advanced uses in clinical dentistry due to the introduction of different laser wavelengths, methods, and delivery systems, which can be used in endodontic treatments to disinfect the root canal system by activating irrigants to improve their effectiveness and decrease the microbial count (Eymirli et al., 2017). Many lasers have been used in endodontics such as neodymium‐doped yttrium aluminum garnet (Nd:YAG); erbium‐doped yttrium aluminum garnet (Er:YAG); erbium‐ and chromium‐doped yttrium, scandium, gallium, and garnet (Er:Cr:YSGG); holmium:yttrium aluminum garnet (Ho:YAG); and diode lasers (de Souza et al., 2008). Diode lasers, which were recently introduced in endodontics, have wavelengths in the range of 800−1064 nm for dental uses and can penetrate to a depth of 500 µm through dentinal tubules for disinfection of the root canal system (Gutknecht et al., 2004). Moreover, they cause thermal photodisruptive activity in unreachable areas of the root canal complex with optimal antibacterial effects within the dentinal tubules (Kimura et al., 2000; Lagemann et al., 2014; Pirnat, 2007).

Several randomized controlled clinical studies have indicated that using IASs reduced the PP pain compared to conventional irrigation (CI) (Gündoğar et al., 2021; Mittal et al., 2023), so this study aimed to clarify the reflection of improving the properties of the final irrigations on the pain experienced by patients after using three IASs—PUI, XP‐Endo Finisher, and diode laser—in their activation of each irrigant solutions used in maxillary necrotic teeth (NaOCl 5.25%, EDTA 17%, and CHX 2%) to determine the most comfrtable IAS during endodontic treatment to patients. The null hypothesis suspects that there is no significant diference in PP after activation the NaOCL, EDTA, and CHX using PUI, XP Endo‐Finisher, or diode laser.

## 2 MATERIALS & METHODS

### 2.1 Study design, settings, and ethical approval

This double‐blinded randomized clinical trial used a three‐arm parallel superiority design with a 1:1 allocation ratio undertaken from January 2020 to March 2022 at the Endodontic Department of the Faculty of Dentistry, Damascus University, Damascus, Syria. This study adhered to the ethical guidelines of the Declaration of Helsinki and received ethical approval from the Local Research Ethics Committee of the Faculty of Dentistry (Approval No. UDDS‐98‐07022019/SRC‐1734). The project was self‐funded and registered at the ISRCTN registry under ID number ISRCTN99457940.

### 2.2 Sample size calculation

The sample size was estimated using G* Power 3.1.9.4 (Heinrich‐Heine‐Universität) based on the changes in visual analog scale (VAS) values. A minimum total sample size of 60 patients (20 in each group) was found to be sufficient for a level of significance of 0.05, a power of 95%, and an effect size (*f*) of 0.535, using values given in a previous paper (Tolibah et al., 2022).

### 2.3 Recruitment and eligibility criteria

One‐hundred and ninety‐four patients aged between 25 and 44 years were referred to the Endodontic Department during the study period because of the presence of apical lesions in their teeth. The patients were investigated by the principal investigator (M. T. A.), where patients' medical and dental history was reviewed. The principal investigator searched for patients with signs of asymptomatic periapical periodontitis such as fistulas and minor percussion pain, or large untreated caries on one or more maxillary incisors (central or lateral). These patients were radiographed to ensure the exist of S3 periapical lesions (large‐sized periapical lesions, >5 mm) according to Venskutonis classification (Venskutonis et al., 2015), the incisor anatomy, the apex diameter, and the cause of the periapical lesion to determine the included incisors. One‐hundred and seven patients met these inclusion criteria. Forty‐seven patients were excluded due to the presence of systemic diseases that compromised general immune status, pregnant females, pateients with preoperative anxiety, psychologically disturbed patients, patients permanently taking analgesic drugs due to chronic systematic conditions, un‐restorable incisors, open‐apex incisors, incisors with multicanals, internal or external resorptions, patients with advanced periodontitis (more than 5 mm periodontal attachment and bone loss), incisors with an unsuccessful previous endodontic treatment, or incisors that were unsuitable for single‐visit treatment, containing moist canals with exudation or pus.

Finally, 60 patients were included in the current study. All included patients who agreed to participate in the study signed an informed‐consent sheet after hearing all of the details about the trial and its therapeutic nature.

### 2.4 Randomization

Incisors were assigned to the PUI group, the XP‐Endo Finisher group, or the diode laser group using the simple randomization method at an allocation ratio of 1:1:1, and a random sequence was created using the website www.random.org, which was accessed on January 1, 2020.

Cards with different irrigant activation methods were placed into opaque and sealed envelopes (20 envelopes per study group), and the patients were instructed to randomly select an envelope. Thus, patients were assigned to three groups: Group 1: PUI as an IAS (*n* = 20), Group 2: XP‐Endo Finisher file as an IAS (*n* = 20), and Group 3: diode laser tip as an IAS (*n* = 20).

### 2.5 Blinding

As the current study was an interventional study, the treating clinician could not be blinded to the type of irrigant activation method used during treatment. However, the patients involved in this study were completely blinded. The assessment of the treatment outcomes was completed by two trained PhD student researchers who were calibrated to the evaluation criteria and blinded to the type of irrigant activation method used.

### 2.6 Clinical procedure

Before anesthesia, the preoperative pain was recorded using a VAS for each included teeth. All caries and previous restorations were removed from the upper incisors under local anesthesia (Huons Lidocaine HCL) and rubber dam (Sanctuary) isolation. The access cavity was refined using an Endo‐Z bur (Dentsply Maillefer), then irrigated with 2 mm of 5.25% NaOCl. The canal orifice was prepared using an orifice opener file (Orodeka Ltd). The working length was determined with an apex locator (C‐smart‐1; COXO) using a K‐file #10 and confirmed by a radiograph.

After achieving the glide path, the canal was shaped using Plex V ORODEKA rotary files (Orodeka Ltd) up to size 25/06 or 35/06 according to the diameter of the apical constriction of the included teeth. The canal was irrigated copiously with 2 mm of 5.25% NaOCl (Merck) using a 30‐gauge endodontic irrigating needle (Sybron Endo, Crop) between files. After instrumentation, the canal was filled with NaOCl 5.25% (Merck). Then, the samples were divided into groups 1−3 according to irrigant activation method.

In Group 1, intermittent ultrasonic irrigation was used, where a #25 U‐file ultrasonic tip (U‐file; Zipperer Co.), mounted on an ultrasonic handpiece (Woodpeker), was inserted inside the filled canal 2 mm before the apex without wall contact. The NaOCl were activated for 45 s at the medium power setting (30 kHz) with a push‐and‐pull movement for each 2 mL of irrigant, which was repeated until achieving 40 mL of NaOCl within 15 min in total.

The method of NaOCl activation with PUI was similar to the described by (Liapis et al., 2021).

In Group 2, the XP‐Endo Finisher file (FKG Dentair) was inserted 2 mm before the apex at the center of the canal and operated at a speed of 800 rpm and a torque of 1 N·cm. Then, it was passed through the entire perimeter of the canal wall to ensure that it was touching larger areas of the root wall and removing the remaining preparation debris. The irrigants were activated for 45 s (2 mL at a time), which was repeated until achieving 40 mL of NaOCl within 15 min in total.

The method of NaOCl activation with XP‐Endo Finisher was similar to the one described by (Nangia et al., 2020).

In Group 3, the laser irradiation was performed using an 810 nm diode laser (Mercury G10; Wuhan Pioon Technology Co., Ltd) coupled with an optical fiber tip (200 µm). The tip was inserted 2 mm before the apex and operated at the following settings: an average power of 1.2 W with a lower frequency of 50 Hz and an energy of 12 J (each cycle) in pulsed mode. Then, the tip was removed using a slow helical movement in the apicoronal direction, irradiated for 10 s, followed by a 10 s pause, which constituted one cycle. Irrigant was activated in increments of 2 mL using four cycles, which was repeated until 40 mL of NaOCl was activated within about 15 min in total.

The method of NaOCl activation with diode laser was similar to the one described by Coelho et al. (2019) and Kaplan et al. (2022).

**Figure 1:**
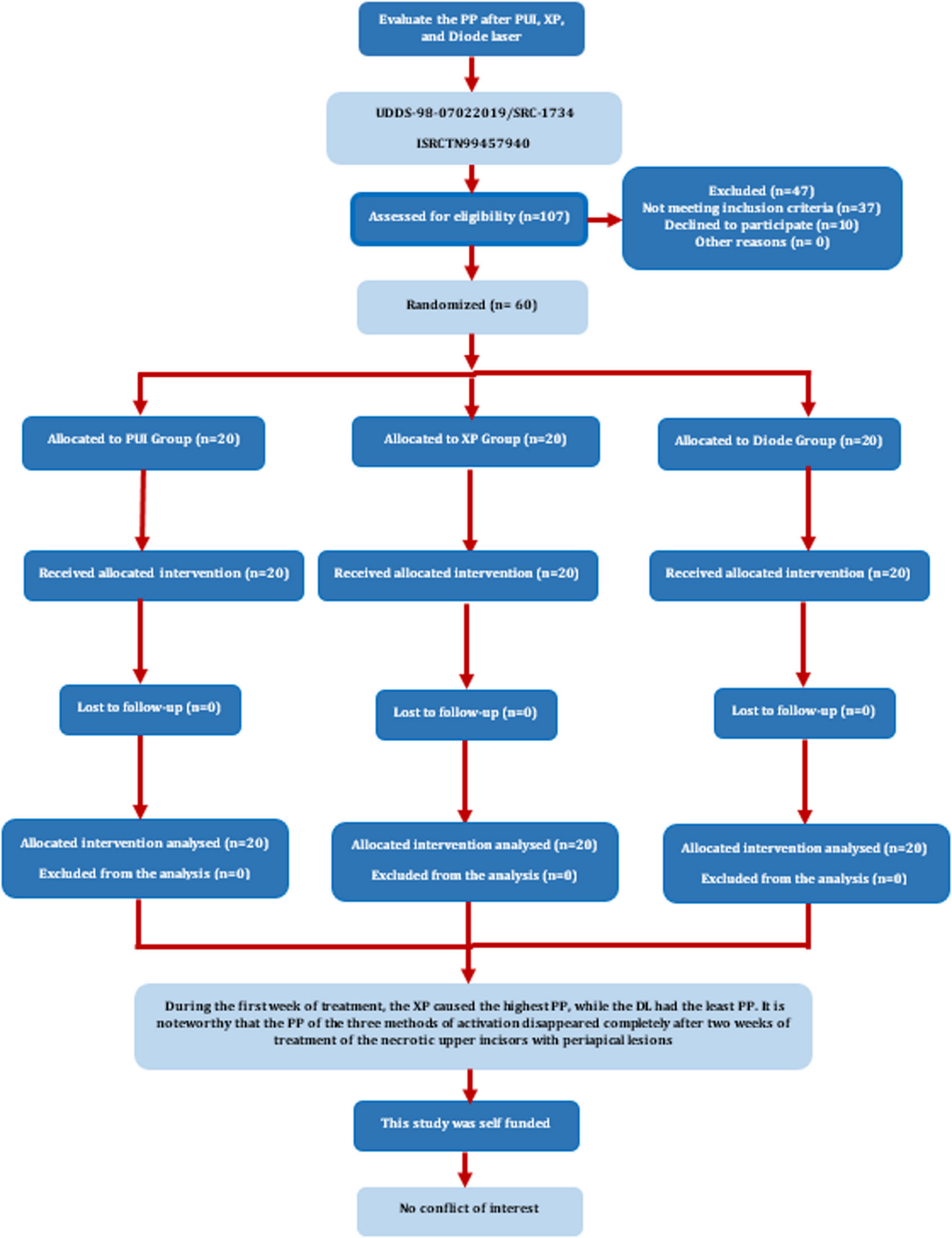
Flow chart of the study. DL, diode laser; PP, postoperative pain; PUI, passive ultrasonic irrigation; XP, XP‐Endo Finisher.

All canals in each group were irrigated with normal saline, filled with 2 mm of EDTA 17% (Produits Dentaires SA), and activated in the same method as that of NaOCl for 15 s, and the procedure was repeated twice. Then, all canals in each group were irrigated with normal saline, filled with 2 mm of CHX 2% (Maquira), and activated in the same method as that of NaOCl and EDTA for 15 s, and that was repeated twice. The method of EDTA and CHX activation was similar to the method described by Prada et al. (2019). Finally, all canals were dried using sterilized paper points (Gabadent) and obturated carefully using gutta‐percha (Gabadent) and AH Plus sealer (Dentsply Sirona) using the warm vertical compaction technique. It was confirmed that there was no overfilling using a radiograph. The canals were restored with suitable resin‐bonded restorations. Patients were asked to take Acetaminophen as an analgesic in case of continuous unbearable pain. A patient flowchart of this study is provided in Figure 1.

### 2.7 Outcomes measures

Patients of all groups were recalled at next day, 3 days, 1 week, and 2 weeks of treatment, and PP was recorded using VAS scale, where patients chose their pain levels by pointing along a 10 cm continuous line between two endpoints, ranging from the absence of pain to unbearable pain.

### 2.8 Statistical analysis

The collected data were tabulated and analyzed using SPSS software (Version 20, IBM SPSS Inc.). Shapiro−Wilk test was used to evaluate if the quantitative measurements showed normal distribution, so the comparison between groups regarding PP was performed using the Kruskal−Wallis test, and the pairwise comparisons were performed using the Mann−Whitney *U* test. The level of significance was set at 0.05.

## 3 RESULTS

A total of 60 necrotic incisors in 60 patients (32 men and 28 women) aged between 25 and 44 years ($\bar{x}$ = 27.1) were included in the study. No significant differences were reported between the groups regarding the age (*p* = 0.531) and gender (*p* = 1.000) of the treated patient, indicating that the allocation of patients into various groups was randomized. It is noteworthy that no patient told that he took any kind of analgesics during the follow‐up periods.

**Figure 2:**
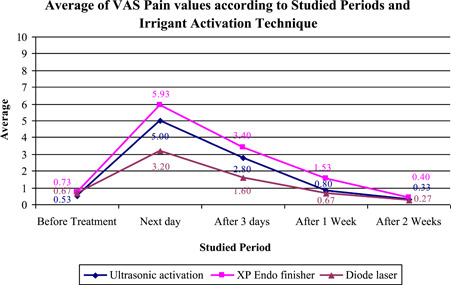
Diagram of pain scores across different follow‐up periods. VAS, visual analog scale.

Table 1 and Figure 2 summarize the mean, standard deviation, range, and Kruskal−Wallis test results of the VAS scores of preoperative pain and PP after 1, 3, 7, and 14 days of treatment in the three groups. The Kruskal−Wallis test showed a significant difference between the groups (PUI, XP–Endo finisher, and diode laser) the next day, 3 days, and 1 week of treatment (*p* = 0.002, *p* = 0.017, and *p* = 0.006, respectively), meanwhile there were no significant differences between the groups before treatment and after 2 weeks of treatment (*p* = 0.616 and *p* = 0.746, respectively).

**Table 1:** Descriptive statistics of the VAS scores and the *p*‐values of significance testing for all three groups.

Studied period	Irrigant activation technique	Number	Mean ± standard deviation	Range	*p* Value^a^
Before treatment	Passive ultrasonic irrigation	20	0.53 ± 0.52	0−1	0.616
	XP‐Endo Finisher	20	0.73 ± 0.59	0−2	
	Diode laser	20	0.67 ± 0.49	0−1	
Next day	Passive ultrasonic irrigation	20	5.00 ± 2.14	3−9	0.002^b^
	XP‐Endo Finisher	20	5.93 ± 2.37	3−9	
	Diode laser	20	3.20 ± 1.26	2−6	
After 3 days	Passive ultrasonic irrigation	20	2.80 ± 1.86	1−6	0.017^b^
	XP‐Endo Finisher	20	3.40 ± 1.88	1−6	
	Diode laser	20	1.60 ± 0.91	1−4	
After 1 week	Passive ultrasonic irrigation	20	0.80 ± 0.86	0−3	0.006^b^
	XP‐Endo Finisher	20	1.53 ± 0.83	1−4	
	Diode laser	20	0.67 ± 0.62	0−2	
After 2 weeks	Passive ultrasonic irrigation	20	0.33 ± 0.49	0−1	0.746
	XP‐Endo Finisher	20	0.40 ± 0.51	0−1	
	Diode laser	20	0.27 ± 0.46	0−1	

Abbreviation: VAS, visual analog scale.

^a^ Kruskal−Wallis test.

^b^ Significant difference.

The Mann−Whitney *U* test was used to detect differences in pairwise comparisons, which showed that, on the next day of the treatment, the diode laser group had the lowest mean VAS score($\bar{x}$ = 3.20) compared with that of the ultrasonic and XP‐Endo Finisher groups, and the differences were statistically significant ($\bar{x}$ = 5.00 and $\bar{x}$ = 5.93; *p* = 0.007 and *p* = 0.001, respectively). However, after 3 days of treatment, the diode laser group had a lower mean VAS score ($\bar{x}$ = 1.60) than the XP‐Endo Finisher group only, and the difference was statistically significant ($\bar{x}$ = 3.40; *p* = 0.005). Finally, after 1 week of treatment, the XP‐Endo Finisher group had a higher mean VAS score ($\bar{x}$ = 1.53) than the diode laser and ultrasonic groups, and the differences were statistically significant ($\bar{x}$ = 0.67 and $\bar{x}$ = 0.80; *p* = 0.003 and *p* = 0.012, respectively).

## 4 DISCUSSION

The first choice to manage apical periodontitis is conservative endodontic treatment, as many reports have indicated that a high proportion of necrotic teeth with apical lesions were healed by the conventional endodontic treatment (Riis et al., 2018; Venskutonis et al., 2015). There are many factors related to root canal treatment that may cause PP; irrigants used during endodontic treatment have toxic effects on vital periodontal tissues, potentially leading to chemical periodontitis, which manifests as severe pain, hemolysis, edema, bleeding from the root canal, and ulceration (Pashley et al., 1985). Although the extrusion of a large amount of irrigant is considered a rare case (Spencer et al., 2007), a low amount of irrigant that triggers the periradicular (Gondim, 2010) and the extrusion of the infected tissues and root canal preparation debris out of the apex during treatment might explain PP (Siqueira & Barnett, 2004; Sipavičiūtė & Manelienė, 2014). Although teeth with apical lesions are more susceptible to flare‐ups after endodontic treatment (Walton, 2002), single‐visit treatment does not increase PP (Patil, 2016).

IASs aim to increase the effectiveness of irrigants in sterilizing the root canal system, which may help to complete the entirety of endodontic treatment of necrotic teeth in single‐visit treatment (Plotino et al., 2016; Verma et al., 2020). However, the use of an IAS may increase the probability of irrigants and preparation debris extrusion beyond the apex (Desai & Himel, 2009; Hizarci et al., 2019), which may irritate the periapical area and increase PP during the first hours after treatment (Gündoğar et al., 2021).

The issue of post‐endodontic pain using CI or an IAS is a controversy. Some studies indicated that IAS may relieve PP in comparison with CI (Elzainy et al., 2022; Erkan et al., 2022). On the other hand, some studies did not find differences in PP levels between IAS and CI (Ali, 2022; Sobhy et al., 2022). Although CI could be a control group, the number of participants in the current study didn't help to increase the groups of the study. Moreover, this study is the first randomized clinical trial that evaluated PP after the combined activation of three final irrigants (NaOCl, EDTA, and CHX) using different activation methods (PUI, XP‐Endo Finisher, and diode laser). Additionally, to the best of our knowledge, there was no study that focused on the treatment of necrotic teeth with large sized apical lesion with single visit tretament.

Patients who used any analgesic that was not related to any dental problem and those having a previous history of pain were not included to reduce all potential preoperative factors. Moreover, patients with diffuse pain were not included in this study, as pain in one tooth may affect the other teeth (Ramamoorthi et al., 2015). Thus, this study was carried out on healthy participants having asymptomatic periodontitis in maxillary incisors with a large‐sized periapical lesion. The main objective of this study was to evaluate PP after the final irrigants activation for necrotic teeth treatment to find the least painful protocol to be adopted in the single‐visit treatments of the necrotic maxillary incisors to make endodontic treatments more comfortable and acceptable for patients.

Continues replenishment during the activation of the three irrigants used are mandatory to maintain their efficacy (Moorer & Wesselink, 1982; Zehnder, 2006). It is worth noting that the irrigation protocols are very different through in vivo studies due to many available irrigation procedures and tooth statue (Tonini et al., 2022). For example, the activation time and NaOCl concentration and total volume was increased to make the irrigation protocol suitable for the treatment of necrotic teeth with large‐sized periapical lesions in single visit treatment. Moreover, the modifications in the irrigation protocol came to make the times of the activation cycles uniform among the three groups as much as possible to investigate the effect of the IAS on PP.

A very careful obturation procedure was adopted to prevent excessive amount of materials (gutta‐percha and sealers) beyond the apex, where cases in all groups received excellent filling at the apex determined by the Apex Locator device and confirmed radially without any increase. Since this factor is controversial for the PP, some studies have found an association between increased pain levels and overfilling (Shashirekha et al., 2018), and others showed no association (Fonseca et al., 2012).

A VAS was used in this study to evaluate PP after the root canal treatment because it is an easy numerical rating scale that has high reliability in acute pain assessments (Bijur et al., 2001).

Previous studies demonstrated that age, gender, and patient preoperative anxiety have a role in PP (Vaughn et al., 2007; Wang et al., 2010). However, in the current study, *p*‐values for age and gender showed that the allocation of patients into various groups was randomized, which means that patient sample had similar distributions among the three groups. Therefore, the effect of these variables was ignored in accordance with previous studies (Ali, 2022; Alves, 2010). Moreover, patients with preoperative anxiety were excluded.

According to the results of this study, the XP‐Endo Finisher activation caused the most PP for a longer period than that of the other IASs. This result might be due to the unique functioning of the XP‐Endo Finisher file, where the file assumes a sickle shape in the root canal, causing more disturbance and pressure, thereby increasing the debris and irrigant amount that extrudes to the periapical area (Nangia et al., 2020). Moreover, the study of Hanafay et al. (2020) met the previous result.

The intermittent ultrasonic irrigation technique was used in the PUI group, where a syringe was used to transfer the irrigants into the root canal. This method allows for controlling of the amount of irrigants used, as well as the penetration depth inside the root canal (Nicoletti et al., 2009). The PUI mechanism comprises of its ability to form cavitation bubbles and cause acoustic streaming, which generates hydrodynamic and shear forces in the irrigants (Keles et al., 2016). Based on our observations, it seemed that the irrigants' movement initiated by the U‐files was less than that by the XP‐Endo Finisher files, as the movement conducted by the latter was more chaotic, which might explain the faster decrease in pain during the third and seventh days of treatment compared to the XP‐Endo Finisher.

Direct laser light emission in a root canal that is filled with an irrigant can generate two effects: the irrigant absorbs the laser energy and forms bubbles, and the collapsed bubbles then create acoustic streaming. Moreover, the photothermal property of lasers in the root canal can increase the temperature of irrigants at any concentration, which reduces their viscosity and improves their ability to penetrate the root dentin (Pradhan & Karnik, 2011; Rooney et al., 1994).

Acoustic cavitation that generate by ultrasonic or diode lasers could have several physical and chemical effects. The oscillation and collapse of cavitation bubbles can generate strong shear force, microjets, microstreaming, and shockwaves in the irrigant. Such strong physical forces have been used in root canal disinfection and deactivate pathogens themselves. Moreover, some evidence suggested that the chemical effects (radicals) of acoustic cavitation are also effective in deactivating pathogens, were the strong oxidizing agents produced within acoustic cavitation bubbles could be used to degrade organic pollutants and convert toxic inorganic pollutants to less harmful substances (Yusof et al., 2016).

A diode laser was adopted in this trial due to the small space occupied by the device, its low cost, and the unique design of the fiber tip. These factors enabled the specialists to use the laser in narrow and curved canals (Lagemann et al., 2014). Although some lasers used in irrigant activation have mechanical‐based activation, in this study, the diode lasers had a greater thermal effect on the irrigants, which caused a minimal extrusion of the irrigant beyond the apex. This factor might explain the decreased PP on the first, third, and seventh day after treatment.

In conclusion, our results showed that the null hypothesis is true just after 2 weeks of treatment, meanwhile the alternative hypothesis is true during the first week of treatment, where the XP‐Endo Finisher group showed the highest level of PP, whereas the diode laser group showed the least level of PP especially at the next day and 3 days of treatment.

Erkan et al. (2022) evaluated different IASs, including laser IAS (shock wave–enhanced emission photo‐acoustic streaming) with Er‐YAG, PUI, and manual dynamic activation with a master gutta‐percha cone in NaOCl activation after chemo‐mechanical preparation; they found that laser stimulation based on heating the irrigant led to significantly lower VAS scores than ultrasonic stimulation and mechanical systems after 2 and 7 days of treatment. The current study had similar findings in the activation of triple irrigants.

In contrast, Omar et al. (2020) made an RCT that compared PP after activating 2.5% NaOCl alone with the 970 nm diode laser and PUI using U‐files after 1, 2, and 3 days of treatment; they concluded that there were no significant differences in VAS scores between these groups. Although the previous study used different methods and IASs, it concluded that there were no differences between mechanical‐ and thermal‐based activation regarding PP. Furthermore, Sobhy et al. (2022) used an 810 nm diode laser in continuous‐wave mode to disinfect the root canal system, but they did not find any significant differences in VAS score after 3 days of treatment compared to using saline alone as a final irrigant—in contrast with the results of the current study. Moreover, Ali and colleagues evaluated the PP following endodontic treatment of molars with symptomatic irreversible pulpitis within a single visit treatment, where a standardized endodontic treatment was performed, followed by either traditional side‐vented needle irrigation, XP‐Endo Finisher, or ultrasonic as an IAS of the final irrigant 2.5% NaOCl. This previous study concluded that there were no statistically significant differences between the previous groups. The reason for the difference between the current study and the previous study may be due to the fact that in the current study, irrigation was activated with longer times of the three irrigants (5.25% NaOCl, 17% EDTA, and 2% CHX) that were used in the case of necrotic teeth with periapical lesions (Ali, 2022).

Despite our best attempts to standardize the criteria of the patients included in the current study, there were problems represented in the pain of the infiltration anesthesia and gingival pain resulting from the rubber dam clamp, which may have affected pain assessment, especially on the next day after treatment. Moreover, since the previous study was a clinical study, it was not possible to standardize the apical diameters of the teeth included in the patients, so we only relied on expanding the apical foramen to three measurements of its basic diameter, and therefore the amount of expansion was not associated with the severity of PP. In addition, not all cases of asymptomatic necrotic incisors were suitable for single‐visit treatment, as some cases were not filled in the same session because the canals were not dried; these cases required a calcium hydroxide dressing that could mask the pain caused by irrigants activation (Kaplan et al., 2022), which forced us to exclude these cases from the current study.

Further studies should be performed on other IASs—with other laser types, sonic irrigation, and manual activation—to determine the most comfortable protocol for patients. Moreover, further studies are required to assess the relationship between the IAS used and periapical lesion healing.

## 5 CONCLUSION

Within the above‐mentioned limitations of this randomized clinical trial, the results showed that IASs had different effects on PP during the single visit treatment of necrotic maxillary incisors within the first week after endodontic treatment: the diode laser group showed the lowest PP levels, whereas the XP‐Endo Finisher group showed the highest PP levels, especially within the first 3 days of treatment. Moreover, the levels of PP after 1 and 2 weeks were low and neither of the IASs (PUI, XP‐Endo Finisher, and diode laser) resulted in any negative effects, and the pain completely disappeared after 2 weeks of treatment.

## AUTHOR CONTRIBUTIONS

Mohammad Tamer Abbara and Ghina Mahayni conceptualized the idea, provided the treatment, and contributed to the writing and documenting. Samar Akil and Omar Hamadah conceptualized the idea and supervised the PhD thesis for Mohammad Tamer Abbara. Yasser Alsayed Tolibah contributed to the interpretation of data and the revision, formatting, and re‐editing of the manuscript. All authors read and agreed to the published version of the manuscript.

## CONFLICT OF INTEREST STATEMENT

The authors declare no conflict of interest.

## ETHICS STATEMENT

The study was conducted according to the guidelines of the Declaration of Helsinki, and was approved by the Local Research Ethics Committee of the Faculty of Dentistry, Damascus University (UDDS‐98‐07022019/SRC‐1734). Informed consent was obtained from all subjects/caregivers involved in the study.

## Data Availability

Deidentified data are available upon written request to the corresponding author.
